# Circulating Plasma microRNA to Differentiate Cushing's Disease From Ectopic ACTH Syndrome

**DOI:** 10.3389/fendo.2020.00331

**Published:** 2020-06-05

**Authors:** Zhanna Belaya, Patimat Khandaeva, Larisa Nonn, Alexey Nikitin, Alexander Solodovnikov, Ivan Sitkin, Andrey Grigoriev, Mikhail Pikunov, Anastasia Lapshina, Liudmila Rozhinskaya, Galina Melnichenko, Ivan Dedov

**Affiliations:** ^1^The National Medical Research Centre for Endocrinology, Moscow, Russia; ^2^Department Pathology College of Medicine, University of Illinois at Chicago, Chicago, IL, United States; ^3^Federal Research and Clinical Center FMBA of Russia, Moscow, Russia; ^4^Ural State Medical Academy, Yekaterinburg, Russia; ^5^National Medical Research Center of Surgery Named After A.V. Vishnevsky, Moscow, Russia

**Keywords:** circulating microRNA, Cushing's disease, ectopic ACTH syndrome, microRNA-16-5p, ACTH dependent Cushing's syndrome

## Abstract

Corticotropinomas and adrenocorticotropic hormone (ACTH)-secreting neuroendocrine tumors exhibit differential levels of some microRNAs (miRs) compared to normal tissue. Because miRs can be released from tissues into circulation, they offer promise as novel disease biomarkers.

**Objective:** To evaluate whether miRs are differentially detected in plasma samples of patients with ACTH-dependent Cushing's syndrome (CS).

**Design:** Case-control study.

**Methods:** Morning fasting plasma samples were collected from 41 consecutive patients with confirmed ACTH-dependent CS and 11 healthy subjects and stored at −80°C. Twenty-one miRs previously reported to be differentially expressed in ACTH-secreting tumors vs. healthy tissue samples were quantified in plasma by qPCR.

**Results:** Among enrolled subjects, 28 were confirmed to have Cushing's disease (CD), 13 had ectopic ACTH secretion (EAS) and 11 were healthy controls. We found statistically significant differences in the circulating levels of miR-16-5p [45.04 (95% CI 28.77–61.31) in CD vs. 5.26 (2.65–7.87) in EAS, *P* < 0.001; *q* = 0.001], miR-145-5p [0.097 (0.027–0.167) in CD vs. undetectable levels in EAS, *P* = 0.008; *q* = 0.087] and differences in miR-7g-5p [1.842 (1.283–2.400) in CD vs. 0.847 (0.187–1.507) in EAS, *P* = 0.02; *q* = 0.14]. The area under the receiver-operator (ROC) curve was 0.879 (95% CI 0.770–0.987), *p* < 0.001, when using miR-16-5p to distinguish between CD and EAS. Circulating levels of miR-16-5p in the healthy control group differed from that of both the CD and EAS groups.

**Conclusions:** Plasma miR levels differ in patients with CD and EAS. In particular, miR-16-5p, miR-145-5p and miR-7g-5p are promising biomarkers for further research to differentiate ACTH-dependent CS.

## Introduction

Cushing's syndrome (CS) is a potentially lethal disease caused by the overproduction of cortisol, most commonly due to adrenocorticotropic hormone (ACTH)-producing pituitary adenomas [Cushing's disease (CD)], or less commonly by either ACTH-producing carcinoid tumors [ectopic ACTH secretion (EAS)] or primary adrenal gland disease (1).

Endogenous CS is a rare disease with an incidence of around 2.4 cases per million per year (2). In ACTH-dependent CS, CD accounts for 80% of cases while EAS accounts for 15–20% (2). Differential diagnosis of ACTH-dependent CS is a challenging clinical issue, as up to 20% of ACTH-secreting pituitary adenomas are not visualized by magnetic resonance imaging (MRI) with gadolinium and the sensitivity of other methods to detect ACTH-producing carcinoid tumors after the first visualization is no more than 43% (3). Additionally, up to 16% of the general population may have a non-functioning pituitary microadenoma of <6 mm and, therefore, there is a probability of a co-accidental non-functioning pituitary microadenoma existing in a patient with EAS (4, 5). Bilateral inferior petrosal sinus sampling (BIPSS) is the gold standard to distinguish between the two forms of ACTH-dependent CS (6). However, the BIPSS procedure is invasive, costly and has certain contraindications and potential complications (7). Blood-circulating microRNAs (miRs) are current promising biomarkers in cancer diagnosis and monitoring (8, 9) as well as in some metabolic diseases (10–12).

MicroRNA (miR) are members of a large class of non-coding RNAs. They work as post-transcriptional gene regulators by binding to the 3′UTR or 5′UTR regions or to the coding sequence, which usually leads to negative expression regulation or exonucleolytic mRNA cleavage. Rarely, positive expression regulation occurs, or a miR can serve as a transcription factor when it is re-localized in the nucleus (13). MiR can be released and encapsulated in the cell-free lipid carriers, be bound to protein complexes with Argonaute or be bound to high-density lipoproteins, which enables them to remain stable in the circulation (14). In fact, miRs can be actively secreted and work as mediators of intercellular and intratissue communication (15).

MiR expression is tissue-specific and shows unique patterns in different cancer tissues. Differential expression of miR-145, miR-21, miR-141, let-7a, miR-150, miR-15a, miR-16, and miR-143 is observed in ACTH-secreting pituitary tumors compared to normal pituitary tissues (16). Moreover, some miRs have been identified as predictors of pituitary adenoma histotypes (17) and pituitary adenoma malignancy (18). Recent reviews have extensively summarized miRs and target genes in various types of pituitary adenomas (19, 20). Several studies reported a difference in miR expression in neuroendocrine tumors including lung carcinoid tumors (21–24). As the profile of miR expression is tumor-specific, it might be possible to distinguish ACTH-dependent CS using circulating miRs as biomarkers.

The goal of this study is to assess differences in circulating levels of preselected miRs in patients with ACTH-dependent CS caused by either CD or EAS.

## Materials and Methods

### Subjects

Forty-one consecutive patients with clinically evident and biochemically proven ACTH-dependent CS, with either a non-visualized pituitary adenoma or an adenoma of <10 mm, and eleven healthy subjects were enrolled in the study. All enrolled subjects provided written informed consent. Patients with ACTH-dependent CS were confirmed to be positive for endogenous hypercortisolism in at least three of the following tests: 24-h urinary free cortisol (24hUFC), serum cortisol after low-dose dexamethasone suppression, late-night salivary cortisol (LNSC), and awake serum cortisol at 23:00 h. Morning and evening ACTH levels were above or within the reference range in all subjects.

Healthy volunteers were recruited from staff and the faculty population of NMRCE. Healthy volunteers did not have any complaints regarding their health, had regular preventive examinations that certified them as generally healthy and did not have signs or symptoms indicative of CS after examination by an experienced endocrinologist.

Exclusion criteria for all participants were pregnancy, glucocorticoid use, alcohol abuse, exacerbation of a chronic disease, severe conditions (i.e., renal and liver insufficiency, heart attack and stroke), terminal conditions, mental insanity, prolonged immobilization (>1 week) and treatment to resolve hypercortisolism.

### Sample Collection and RNA Isolation

All subjects had fasting blood samples collected at 08:00–09:00 a.m. into EDTA tubes. Samples were centrifuged twice at +5°C and 3,000 rpm for 20 min, then aliquoted and stored at −80°C until further analysis. MiRNA isolation from plasma was carried out with a miRNeasy Serum/Plasma Kit (Qiagen) on the automatic “QIAcube” station per the manufacturer's protocol. To prevent degradation, 1 unit of RiboLock RNase Inhibitor (Thermo Fisher Scientific) was added per 1 μL of RNA solution. The concentration of miRNA in the aqueous solution was evaluated on a NanoVue Plus spectrophotometer (GE Healthcare).

### Quantification of miR Transcripts by qRT-PCR

Twenty-one miRs previously reported to be differentially expressed in ACTH-secreting tumors compared with healthy tissue samples (hsa-miR-10b-5p, hsa-miR-129-5p, hsa-miR-133a-5p, hsa-miR-141-3p, hsa-miR-143-3p, hsa-miR-145-5p, hsa-miR-150-3p, hsa-miR-15a-5p, hsa-miR-16-5p, hsa-miR-146a-5p, hsa-miR-185-3p, hsa-miR-191-5p, hsa-miR-203a-5p, hsa-miR-210-5p, hsa-miR-211-5p, hsa-miR-31-5p, hsa-miR-409-3p, hsa-miR-−409-5p, hsa-miR-431-5p, hsa-miR-488-3p, and hsa-miR-7g-5p) were quantified in the plasma by qPCR (Supplementary Table 1).

qRT-PCR was performed using a TaqMan Advanced miRNA cDNA Synthesis Kit (Thermo Fisher, A28007) and TaqMan® Advanced miR Assays (Thermo Fisher Scientific, A25576), and run in a 96-well format on the StepOnePlus instrument (Applied Biosystems) according to the manufacturer's protocol. All qRT-PCR reactions were performed in 3 technical replicates for each sample. Data analyses were performed using SDS software (version 2.3, Applied Biosystems), and a comparative C_T_ method (ΔC_T_) was used for the relative quantification of miR circulating levels. We used value Ct <35 as a cutoff of detection. All samples were normalized to hsa-miR-191 levels and spike-in control cel-miR-39-3p, hsa-miR-191 was selected from recommended list of stably expressed miRNAs in serum and plasma by Thermo Fisher Scientific (hsa-miR-24, hsa-miR-484, hsa-miR-93-5p, hsa-miR-191-5p, hsa-miR-126-3p, hsa-miR-16-5p) as the most stable normalizer. For technical normalization of RT-qPCR, cel-miR-39-3p was added to plasma during the lysis step of miRNA extraction at approximately 10 pM final concentration.

### Routine Measurements

Subjects also provided morning and late-night blood and saliva samples for routine hormonal status measurements: ACTH (reference range: morning 7–66 pg/mL, late-night 0–30 pg/mL), morning cortisol (reference range 123–626 nmol/L), awake late-night serum cortisol at 23:00 h (reference range 46–270 nmol/L), serum cortisol after a low-dose dexamethasone suppression test (cutoff value for suppression, 50 nmol/L) (1), late-night salivary cortisol (reference range 0.5–9.4 nmol/L) (25), and prolactin (reference range 90–540 mIU/L). ACTH, prolactin, and cortisol in the serum and saliva were assayed by electrochemiluminescence assay (ECLIA) using a Cobas 6000 Module e601 (Roche). The 24hUFC was measured by an immunochemiluminescence assay (extraction with diethyl ether) on a Vitros ECi (reference range 60–413 nmol/24 h).

MRI was performed using a Siemens Magnetom Harmony 1.0T with Gadolinium. Multispiral computed tomography (CT) was performed using an Aquilion One system (Toshiba Medical Systems Corporation) with Optiray 300. Patients with an occult tumor also underwent gastroscopy, colonoscopy, total body somatostatin-based positron emission tomography, and thyroid and gonad ultrasound.

BIPSS was performed through a percutaneous bilateral approach. Catheters were inserted into the femoral vein and advanced to the petrosal sinuses under fluoroscopic guidance. Once catheters were properly placed, blood samples were withdrawn simultaneously from each petrosal sinus and a peripheral vein. After obtaining three basal samples (two for ACTH and one for prolactin measurements), intravenous injection of 8 mg desmopressin was administered as a bolus and post-stimulation samples were obtained at 3′, 5′, and 10′. All samples were immediately collected in prechilled tubes containing EDTA kept on ice until centrifugation, which occurred within an hour. A central-to-peripheral ACTH maximal ratio ≥2 in the basal condition and ≥3 after stimulation was considered positive for CD. Prolactin measurements were used as additional proof for correct catheter placement. A prolactin ratio ≥2 in the basal condition was obtained in all patients who underwent BIPPS and was considered positive for correct catheter placement to avoid false negative results (26, 27).

### Statistical Analysis

Quantitative data received from the original miR PCR analysis were transformed using log_2_ fold changes (FC) compared to the control (cel-39) to allow direct comparison between the groups. Log_2_ FC values were then compared between each pair of groups using an independent sample *t*-test with calculation of unadjusted *p*-value (*p*) and adjusted q-value for false-discovery rate (q FDR) using the Benjamini-Hochberg correction method.

Means and 95% confidence intervals of log_2_ FC were plotted for better graphical representation of the results. Effect size (log_2_ FC difference between the pairs of groups) was then plotted using a volcano plot against the –log_10_ adjusted *p*-value (i.e., q FDR), using a threshold of 2 for the log_2_ FC difference and 1.0 for the –log10 q FDR value.

The total area under the receiver-operator (ROC) curve (AUC), along with the significance of the difference of the AUC from 0.5, were presented to show the diagnostic performance of the parameters selected, and sensitivity and specificity values were calculated to confirm the performance of the selected cut-off value in the proper classification of cases. The cut-off value was selected based on maximum sensitivity and specificity and presented along with the confidence interval obtained from the confidence region of the ROC curve.

Data are presented using mean (M) values and 95% confidence intervals (95% CI) or median (Me) and interquartile range (Q25–Q75). Comparisons between the descriptive parameters of patients with CD and EAS were made using unpaired, two-sided *t*-tests. Mann-Whitney *U*-test was utilized to compare quantitative parameters in demographic data. Fisher's exact test was used to compare two independent groups for qualitative parameters.

All statistical analysis and graphical output were performed using R software version 3.4.0.

## Results

A total of 52 participants were enrolled in the study: 41 patients with ACTH-dependent CS and 11 healthy subjects in a control group. In patients with ACTH-dependent CS, a pituitary adenoma was visualized in 17 cases. Adenoma size was measured by MRI, and the mean size was 3 mm (min. 3 mm; max. 8 mm). In 32 cases BIPSS was performed: in 24 because of no adenoma on MRI, and in 8 because the adenoma was <6 mm. Patients with CD (*n* = 28) were referred to neurosurgery based on the presence of a benign pituitary adenoma >6 mm on MRI or BIPSS positive for CD. CD was confirmed in all 28 cases based on histological evaluation, and these patients achieved remission after neurosurgery. Thirteen patients with ACTH-dependent CS were classified as EAS based on BIPPS. Lung carcinoid tumors were detected by CT scan in 8 patients with EAS. After surgery, remission was achieved in all cases and a typical lung carcinoid was histologically confirmed. In two cases of EAS confirmed by BIPSS a lung tumor was suspected based on diagnostic scans. However, in these two subjects, no lung surgery was performed due to severe hypercortisolism and uncertainty with regards to the success of lung surgery in these cases. Whole-body CT scans, somatostatin-based positron emission tomography and other tests did not show any abnormalities in 3 cases. A bilateral adrenalectomy was performed on these five patients. The general characteristics of participants are presented in Table 1.

**Table 1:** General characteristics of study subjects.

	**Median (Q25%; Q75%)**			***p***
	**Cushing's disease**	**Ectopic ACTH syndrome**	**Healthy controls**	
*N*	28	13	11	–
Sex F (%): M (%)	24 (86%): 4 (14%)	6 (46%): 7 (54%)	9 (89%): 2 (11%)	0.003
Age	37 (33.1–42.6)	43 (33.8–52.1)	39 (33.9–44.9)	0.5
BMI	29.8 (27.2–32.4)	30.1 (28.8–31.5)	28.2 (22.2–34.1)	0.4
ACTH 09:00 h	66.2 (45.3; 95.5)	150 (127; 206.5)	–	<0.001
ACTH 23:00 h	48.1 (28.7; 73)	133 (112.1; 166)	–	<0.001
24hUFC	1967.6 (1508; 2041)	2554 (1349; 4888)	–	0.04

*F, female; M, male; BMI, body mass index; ACTH, adrenocorticotropic hormone; 24hUFC, 24 h urine free cortisol*.

The circulating levels of 21 miRs that were measured in CD, EAS, and healthy subjects are presented in Table 2. Significant differences were found between patients with CD and those with EAS in the circulating levels of miR-16-5p [45.04 (95% CI 28.77–61.31) in CD vs. 5.26 (2.65–7.87) in EAS, *p* < 0.001; *q* = 0.001], miR-145-5p [0.097 (0.027–0.167) in CD vs. undetectable levels in EAS, *p* = 0.008; *q* = 0.087] and miR7g-5p [1.842 (1.283–2.400) in CD vs. 0.847 (0.187–1.507) in EAS, *p* = 0.02; *q* = 0.14]. Additionaly, we analyzed the difference in these circulating miR levels between CD (*n* = 28) and EAS (*n* = 8) cases whose diagnosis was confirmed by histological evaluation. There were statisically significant differences in the circulating levels of miR-16-5p: 45.04 (95% 28.77–61.31) in CD vs. 3.18 (95% 0.52–5.85) in EAS (*p* = 0.012), but not in miR-145-5p levels (*p* = 0.141) or miR-7g-5p (*p* = 0.051).

**Table 2:** Differential levels of serum miRNAs in patients with Cushing's disease (CD), ectopic ACTH syndrome (EAS) and healthy controls.

**miR**	**CD, M (95% CI) (*n* = 28)**	**EAS, M (95% CI) (*n* = 13)**	**Healthy subjects, M (95% CI) (*n* = 11)**	**CD vs. healthy controls**		**CD vs. EAS**		**EAS vs. healthy controls**	
				***p***	***q***	***p***	***q***	***p***	***q***
miR-10b-5p	0.293 (0.120–0.465)	1.066 (0.625–2.757)	0.195 (0.015–0.376)	0.410	0.717	0.334	0.606	0.280	0.519
miR-129-5p	0.641 (0.367–0.915)	0.361 (0.124–0.847)	0.585 (0.216–1.386)	0.885	0.987	0.288	0.288	0.602	0.665
miR-133a-5p	0.083 (0.088–0.254)	0.000 (0.000–0.000)	0.000 (0.000–0.000)	0.327	0.623	0.327	0.606	0.118	0.413
miR-141-3p	27.693 (14.19–41.18)	50.838(11.61–90.06)	35.031 (9.458–60.604)	0.586	0.879	0.240	0.606	0.555	0.665
miR-143-3p	1.416 (0.764–2.068)	10.015 (11.887–31.91)	1.421 (0.123–2.719)	0.994	0.994	0.402	0.606	0.403	0.567
**miR-145-5p**	**0.097 (0.027–0.167)**	**0.000 (0.000–0.000)**	**0.100 (0.033–0.166)**	**0.948**	**0.994**	**0.008**	**0.087**	**0.007**	**0.078**
miR-150-3p	0.482 (0.140–0.824)	0.778 (0.125–1.680)	0.380 (0.122–0.883)	0.720	0.987	0.511	0.511	0.405	0.567
miR-15a-5p	0.238 (0.050–0.427)	0.208 (0.249–0.665)	0.110 (0.020–0.240)	0.245	0.571	0.896	0.896	0.652	0.665
**miR-16-5p**	**45.036 (28.76–61.308)**	**5.263 (2.654–7.872)**	**24.159 (13.080–35.238)**	**0.032**	**0.223**	**0.000**	**0.001**	**0.003**	**0.073**
miR-146a-5p	0.065 (0.009–0.121)	0.045 (0,002–0.092)	0.027 (0.008–0,045)	0.184	0.483	0.556	0.687	0.435	0.571
miR-185-3p	5.073 (2.885–7.262)	5.570 (0.055–11.195)	2.029 (0.361–3.698)	0.025	0.223	0.859	0.896	0.204	0.508
miR-191-5p	3.921 (1.762–6.081)	4.490 (1.484–7.495)	2.084 (0.324–3.844)	0.171	0.483	0.746	0.746	0.147	0.441
miR-203a-5p	0.000 (0.000–0.000)	0.000 (0.000–0.000)	0.000 (0.000–0.000)	0.884	0.987	0.077	0.232	0.118	0.413
miR-210-5p	0.000 (0.000–0.000)	0.000 (0.000–0.000)	0.000 (0.000–0.000)	0.884	0.987	0.077	0.232	0.118	0.413
miR-211-5p	0.000 (0.000–0.000)	0.000 (0.000–0.000)	0.000 (0.000–0.000)	0.893	0.987	0.060	0.211	0.104	0.413
miR-31-5p	0.000 (0.000–0.000)	0.000 (0.000–0.000)	0.004 (−0.004 to 0.012)	0.322	0.623	0.077	0.232	0.322	0.519
miR-409-3p	0.545 (0.218–0.871)	1.701 (1.239–4.640)	0.281 (0.051–0.511)	0.172	0.483	0.484	0.404	0.308	0.519
miR-409-5p	3.034 (1.296–4.772)	5.173 (1.155–11.501)	1.613 (0.668–2.557)	0.142	0.483	0.879	0.671	0.242	0.508
miR-431-5p	2.407 (1.237–3.578)	2.574 (0.530–4.618)	1.341 (0.530–2.152)	0.123	0.483	0.879	0.896	0.233	0.508
miR-488-3p	0.027 (0.002–0.056)	0.243 (0.295–0.780)	0.087 (0.106–0.280)	0.506	0.818	0.393	0.606	0.312	0.647
**miR-7g-5p**	**1.842 (1.283–2.400)**	**0.847 (0.187–1.507)**	**0.694 (0.288–1.099)**	**0.001**	**0.026**	**0.020**	**0.140**	**0.665**	**0.665**

*M, mean; 95% CI, 95% confidence interval; miR, microRNA; CD, Cushing's disease; EAS, Ectopic ACTH syndrome*.

Circulating levels of miR-16-5p in the healthy control group differed from that of both the CD and EAS groups (Table 2). While the circulating miR-145-5p level differed between EAS and healthy subjects, it was unchanged between CD and healthy subjects. While miR-7g-5p was differentially expressed in patients with CD compared with healthy controls, it was unchanged in patients with EAS compared with healthy subjects (Table 2).

As miR-16-5p showed the most significant difference in circulating levels between CD and EAS, we specifically tested the potential diagnotic performance of this miR. The AUC was 0.879 (95% CI 0.770–0.987, *p* < 0.001) when using miR-16-5p plasma circulating levels to distinguish between CD and EAS. At the cut-off value of dCT = 9.3825 (95% CI: 5.65–11.73), the sensitivity of miR-16-5p was 90.9% (95% CI: 72.73–100.00) and the specificity was 77.8% (95% CI: 62.96–92.59) for the separation of plasma samples obtained from patients with EAS compared to CD.

Several miRs were practically undetectable in plasma samples (miR-133a-5p; miR-31-5p; miR-210-5p; miR-211-5p), whereas some miRs were abundantly expressed (miR-16-5p; miR-185-3p; miR-141-3p; miR-143-3p) (Table 2).

Patients with CD differ from those with EAS in sex distribution, which was evident in our study. As healthy subjects were matched to patients with CD, we also analyzed the differences in miR expression between sexes. miR-145-5p was differentially expressed in male and female subjects (*p* = 0.003, *q* = 0.067; Table 3). Nevertheless, a direct comparison within female subjects in CD (*n* = 28) vs. EAS (*n* = 5) confirmed statistically significant differences between miR-16-5p *p* = 0.007; miR-145-5p *p* < 0.01, but not miR-7g-5p (*p* = 0.239). There weren't statistically significant differences among men, because of the small sample size.

**Table 3:** Sex-biased preselected microRNA (miR) levels.

**miR**	**Men [M (95% CI)] *n* = 11**	**Women [M (95% CI)] *n* = 30**	***p***	***q***
miR-10b-5p	0.156 (0.011–0.301)	0.538 (0.064–1.012)	0.123	0.263
miR-129-5p	0.289 (−0.055 to 0.632)	0.656 (0.361–0.950)	0.095	0.263
miR-133a-5p	0.000 (0.000–0.000)	0.061 (−0.062 to 0.184)	0.324	0.400
miR-141-3p	46.671 (8.018–85.323)	30.601 (19.490–41.712)	0.398	0.440
miR-143-3p	0.725 (−0.202 to 1.653)	4.198 (−1.692 to 10.088)	0.244	0.390
**miR-145-5p**	**0.011 (−0.002 to 0.024)**	**0.094 (0.042–0.146)**	**0.003**	**0.067**
miR-150-3p	0.310 (−0.085 to 0.706)	0.595 (0.248–0.942)	0.260	0.390
miR-15a-5p	0.237 (−0.177 to 0.652)	0.191 (0.052–0.331)	0.822	0.822
miR-16-5p	17.875 (3.201–38.95)	35.814 (24.033–47.59)	0.125	0.263
miR-146a-5p	0.018 (−0.000 to 0.036)	0.063 (0.021–0.105)	0.048	0.216
miR-185-3p	4.079 (−0.165 to 8.323)	4.639 (2.751–6.526)	0.797	0.822
miR-191-5p	1.441 (−0.532 to 3.414)	4.419 (2.734–6.104)	0.021	0.216
miR-203a-5p	0.000 (0.000–0.000)	0.000 (0.000–0.000)	0.215	0.376
miR-210-5p	0.000 (0.000–0.000)	0.000 (0.000–0.000)	0.215	0.376
miR-211-5p	0.000 (0.000–0.000)	0.000 (0.000–0.000)	0.306	0.400
miR-31-5p	0.000 (0.000–0.000)	0.001 (−0.001 to 0.003)	0.305	0.400
miR-409-3p	0.152 (−0.123 to 0.427)	0.937 (0.120–1.754)	0.070	0.244
miR-409-5p	1.281 (−0.217 to 2.779)	3.816 (1.766–5.866)	0.043	0.216
miR-431-5p	1.185 (0.145–2.225)	2.537 (1.575–3.498)	0.051	0.216
miR-488-3p	0.236 (−0.249 to 0.721)	0.041 (−0.014 to 0.097)	0.398	0.440
miR-7g-5p	0.901 (0.237–1.565)	1.510 (1.066–1.954)	0.116	0.263

*M, mean; 95% CI, 95% confidence interval; miR, microRNA; CD, Cushing's disease; EAS, ectopic ACTH-syndrome*.

## Discussion

This is the first study to show different plasma circulating levels of three miRs (miR-16-5, miR 145-5p, and miR 7g-5p) in patients with CD vs. EAS. These miRs may be promising minimally invasive biomarkers to differentiate patients with ACTH-dependent CS. Because miR-16-5p was the most abundantly expressed in plasma samples and therefore potentially the most easily detected, this miR was tested as a diagnostic biomarker and had good diagnostic performance, with an AUC close to 0.9. MiR-16-5p was differentially expressed not only between subjects with CD vs. EAS, but also between healthy subjects and both forms of ACTH-dependent CS. Interestingly, the circulating level of miR-16-5p was upregulated in plasma samples of patients with CD in our study, but was previously reported as down-regulated in ACTH-secreting pituitary adenomas vs. normal tissue (16). Currently we cannot prove the source of the significantly increased circulating level of miR-16-5p in patients with CD. It may be explained either by a higher release from a pituitary adenoma or this miR being produced by another tissue. At the same time, circulating miR-16-5p was down-regulated in patients with EAS compared to both CD and healthy subjects. When overexpressed, miR-16 suppresses cell proliferation and cell cycle progression, acting as a tumor suppressor (28). Renjie et al. found that miR-16 targets the oncogene *SOX5* and suppresses cell proliferation, migration and invasion (29). Further, in another report, oncoprotein BCL2 was postulated as a target of post-transcriptional repression by miR-16 (30).

We also found that in patients with CD, circulating let-7g was upregulated compared to EAS and healthy subjects. HMGA1 and HMGA2, proteins associated with changes in histone tails and a reduction in gene silencing, are targeted by both miR-16 and let-7 (miR7g-5p) (31).

Additionally, circulating miR-145-5p was down-regulated in patients with EAS, but was not differentially detected in CD and healthy subjects. The difference in miR-145-5p circulating levels might be related to sex distribution; 50% of EAS patients were male while most patients with CD were female, and the difference between males and females was statisically significant. According to previous research, miR-145-5p is downregulated 2-fold in ACTH-producing pituitary adenomas compared with normal pituitary tissue (16, 20) and in primary ileac carcinoids compared with metastasis (32). The downregulation of let-7g and miR-145 has been noted in breast cancer (33), lung cancer (34) and colorectal neoplasias (35). miR-145 circulating levels inversely correlate with the expression of corticotroph tumor oncogenes MYC, KRAS, FOS and YES (16). Further, potential target genes of miR-145 encode proteins with oncogenic functions, such as MYC, KRAS, FOS, YES and FLI, as well as cyclin D2 and MAPK (36).

There are a limited number of studies evaluating circulating miR in patients with pituitary adenomas and carcinoid tumors. We are not aware of any studies focused on circulating miRs in EAS or in patients with CD. Nevertheless, circulating miR-182, 196a, 200a, 21-5p, 22-3p, and 7-5p were upregulated and miR-31, 129-5p, 133a, 215, and 150-5p were downregulated in patients with small bowel neuroendocrine tumors (9, 36–38). Downregulation of miR has been observed in plasma samples of patients with pituitary adenoma compared to samples from healthy subjects (39). Circulating levels of 29 miRs and isomiR variants were able to distinguish preoperative plasma samples from normal controls. Exclusively in FSH/LH+ adenomas, miR-143-3p was downregulated in late, but not early post-operative plasma samples compared to preoperative samples. Plasma levels of miR-143-3p discriminated between these samples with 81.8% sensitivity and 72.3% specificity (AUC = 0.79; *p* = 0.02). However, patients with CD were not enrolled in this study (39). Circulating miRs were differently expressed in patients with acromegaly vs. healthy subjects (40).

Our study has potential limitations. This was a discovery cohort and the study needs additional validation in the future. We were not able to histologically confirm the tumor in 5 out of 13 subjects with EAS, which was only confirmed by BIPSS. Occult tumors are expected among patients with EAS and are well-documented (41). Thus, to minimize false-negative results, we measured the prolactin ratio along with ACTH in all subjects to confirm adequate catheter positioning. In addition to this, we separately analyzed the difference in the circulating miR levels of patients whose diagnosis was confirmed by histological evaluation, demonstrating that the difference in miR-16-5p levels remained statistically significant. Nevertheless, different types of neuroendocrine tumors have different miR- expressions. As all of our confirmed causes of EAS were lung carcinoid tumors, the data can be applied to distinguish between CD and EAS caused by lung carcinoids and perhaps indicate occult tumors, but this needs further investigation. We did not measure plasma circulating miR levels after surgery as the goal of this study was to differentiate patients with ACTH-dependent CS, not to predict remission or recurrence. The power for this sample size was not calculated, as this is a pilot study for a rare disease.

In conclusion, our findings of differential circulating levels of miR-16-5p, miR7g-5p, and mir-145-5p in plasma samples of patients with CD compared with EAS suggest that these miRs may be promising biomarkers for further research to differentiate ACTH-dependent CS, in particular CD and EAS caused by lung carcinoids.

## Data Availability Statement

The datasets generated for this study are available on request to the corresponding author.

## Ethics Statement

The studies involving human participants were reviewed and approved by Local Ethical Committee of the National Medical Research Centre for Endocrinology. The patients/participants provided their written informed consent to participate in this study.

## Author Contributions

AN, PK, IS, AG, MP, and AL carried out the experiments. ZB, LN, and LR supervised the experiments. AN and AS analyzed and interpreted the data. PK and ZB wrote the draft. ID and GM critically revised the draft. All authors approved the final version of the submitted manuscript.

## Conflict of Interest

The authors declare that the research was conducted in the absence of any commercial or financial relationships that could be construed as a potential conflict of interest. The handling Editor declared a past co-authorship with one of the author LR.
