# Supplementary material for: Circulating Plasma microRNA to Differentiate Cushing's Disease From Ectopic ACTH Syndrome
Source: Front Endocrinol (Lausanne). 2020 Jun 5;11:331. doi: 10.3389/fendo.2020.00331 (PMC7291947; doi:10.3389/fendo.2020.00331)
Supplement: Supplementary file 1 [file Data_Sheet_1.PDF]

TaqMan® Advanced miRNA Assays

microRNA	Assay ID
hsa-miR-10b-5p	478494_mir
hsa-miR-129-5p	477896_mir
hsa-miR-133a-5p	478511_mir
hsa-miR-141-3p	478501_mir
hsa-miR-143-3p	77912_mir
hsa-miR-15a-5p	477858_mir
hsa-miR-145-5p	477916_mir
hsa-miR-146a-5p	478399_mir
hsa-miR-150-3p	478721_mir
hsa-miR-185-3p	478732_mir
hsa-miR-7g-5p	483061_mir
hsa-miR-203a-5p	478756_mir
hsa-miR-210-5p	478765_mir
hsa-miR-409-5p	478872_mir
hsa-miR-211-5p	478507_mir
hsa-miR-31-5p	478015_mir
hsa-miR-409-3p	478084_mir
hsa-miR-431-5p	478889_mir
hsa-miR-488-3p	478129_mir
hsa-miR-191-5p	477952_mir
hsa-miR-16-5p	477860_mir
cel-miR-39-3p	478293_mir
